# Nurturing the Human Dimension in Digital and Medical Spaces Through Pedagogy of Care – a Case of Creative Enquiry

**DOI:** 10.5334/pme.1147

**Published:** 2024-05-15

**Authors:** Louise Younie, Chie Adachi

**Affiliations:** 1 Medical Education, Faculty of Medicine and Dentistry, Queen Mary University of London, London, United Kingdom; 2 Faculty of Medicine and Dentistry, Queen Mary University of London, London, United Kingdom

## Abstract

The COVID-19 pandemic ushered in digital learning experiences to front and centre of medical education in disruptive ways. As the pandemic subsides students and educators sigh in relief, longing to move away from the loneliness and disconnection and back to the norms of face-to-face learning and consulting. In the field of medical education however, the need for digital education has exponentially increased over the decade with strong evidence for future growth. We face the pressure of increasing student numbers on clinical placement and some students now desire or even need hybrid options for the flexibility of time, place, and pace.

There is persistent criticism that digital education lacks human connection. This paper argues, however, that it is possible and vital to humanise the virtual learning experience, though particular attention needs to be given to digital pedagogy and relational aspects of learning and teaching. Drawing on Noddings’ pedagogies of care and her theoretical model, we unpack one case-study of a medical education elective course that transitioned online during the pandemic. The aim of this paper is to engage medical educators with the *pedagogy of care* and *relational pedagogy* literature, which are currently almost absent from the medical education literature, as applied to the digital education realm. Core themes include modelling care and connection, enabling dialogue, inviting student engagement and practice in caring for each other and supporting the deeper work of being present themselves and confirming each other. Limitations and implications for future research will also be explored.

## Background and Need for Innovation

During the COVID-19 pandemic medical curriculum shifted rapidly from face-to-face to digital spaces. Terms such as panic-gogy [1] and Remote Emergency Teaching [2] capture the lack of considered pedagogical underpinning. In particular, there was often a focus on maintaining ‘continuity’ of instruction and assessment, with less discussion about how we maintain the communities at the heart of our educational institutions [3]. The resulting student and teacher experience was often seen as disconnected and lonely. As the body of existing literature suggests, sense of belonging and engagement are vital to academic and personal success of student life at universities [4].

Although there is a palpable desire to return to face-to-face learning and teaching, multiple forces drive digital approaches in medical education, including the need for learning flexibility in the curriculum, expansion of student numbers for university as a business, and growth of digital health practice in the clinical workplace. How might we create a digital experience that enables connection, belonging and community?

In this paper, drawing on the theoretical framework of Noddings’ pedagogy of care [5,6], we challenge the framing of digital experience as the poor relative and second citizen to face-to-face encounters, and/or ‘less than’ [7]. We propose that engaging online learning communities needs purposeful learning design to create a sense of belonging and human connection and illustrate this with an innovation that emerged in a medical education elective course that rapidly transitioned online during the pandemic.

## Goal of Innovation

The goal of our innovation is to introduce the human dimension into online learning spaces drawing on the concept of pedagogy of care [5]. Our approach of self-study [8] has enabled critical dialogue between the two authors and in-depth analysis of the rapid transition of a medical education elective course as an educational innovation.

### On pedagogy of care

Relational pedagogy more broadly [9] and in particular Noddings’ framework regarding the pedagogy of care [5,6] is largely missing from medical education literature. Noddings conceptualises four key components: *modelling, dialogue, practice* and *confirmation* that underpin relational, ethical and moral principles of education. She argues that both educator and student perspectives are critical to enact the caring ethics and practice of teaching and learning. This work fundamentally follows social constructivist and feminist schools of thought, collectively giving voice and advocacy for moving beyond behaviourist and cognitivist approaches to higher education.

In Noddings work, **modelling**, refers to the role-modelling of care by the teacher. **Dialogue** refers to open and respectful conversations between teachers and students in a trusting environment [5]. **Practice** is where students have the opportunity to practise caring for themselves, their peers and others, for example, in small group work where students learn from each other and solve issues together [5,6]. **Confirmation** refers to enabling students to seek and identify an ethical and attainable self, that is of their ‘better self’, they aspire to be [5]. In the next section, we will illustrate how the transition to online learning and teaching was made successful through practical examples of teaching strategies – a summary of those is provided in the Table 1.

**Table 1:** Summary of teaching strategies used for humanising digital learning spaces.


MODELLING

Connect with students in advance of teaching – e.g. online questionnaire on learning needs or challenges Attention to building relationships and trust, e.g. student identified group rules Introduce online learning principles – e.g. psychological safety, equal talk time, kindness matters Expect presence and screens on for video interaction where possible Carefully plan meaningful and non-threatening learner tasks Enact vulnerable leadership through modelling by the educator

**DIALOGUE**

Shorten any presentation or didactic talking to ten minutes in an hour Enhance informal dialogue – e.g. WhatsApp group for building connection Embody awareness in digital learning – breaks and movement Start/end groups with ‘Kettle boiling’ breakouts to invite voice Invite individual thinking time, small and large group dialogue (think – pair – share)

**PRACTICE**

Enable online sharing of creative enquiry work via digital platforms such as Padlet Attend to/hold emotional space in the online environment

**CONFIRMING**

Enable deep listening, presence, curiosity and diversity of perspectives Enable space for lived experience, transformative engagement


## Steps Taken for Development and Implementation of Innovation

The case we explore in this paper is a Student Selected Component (SSC) ‘Exploring the creative arts in health and illness’ course. It is optional and has been running face-to-face, designed, developed, and led by the first author for twenty years. Groups of up to twelve students experience a range of facilitators, also carefully selected by the first author, including clinicians, arts therapists, patient artists, arts for health consultants, across different creative approaches such as creative writing, photography, drama therapy [10]. Funding follows students undertaking SSCs in the UK although the administration of payment to ad hoc tutors remains challenging.

The course runs predominantly as a series of up to eight, two- or three- hour workshops starting and ending with workshops run by the first author who also maintains oversight and connection with the students throughout the whole two weeks. The learning is underpinned by a creative enquiry pedagogical approach. Creative enquiry is the exploration of lived experience through the arts [11], allowing multi-modal student reflection on experience through any of the different languages of the arts – e.g. poetry, sculpture or dance coupled with reflective writing.

During the pandemic (2020–2022), four courses were facilitated entirely virtually. A total of 47 students participated in the course across four different cohorts of students and the course was similarly run with a series of creative enquiry workshops.

### Modelling

To allow for clarity, a new website was developed with workshop overviews, links to other resources and bibliography. The front page clearly outlines the expectation for online engagement:

*Due to Covid 19, this course will be facilitated virtually and we will meet mainly on Zoom. We expect you to turn up with your videos on as this is essential for the group work. Please can you connect with your laptop if at all possible so that the whole group can see each other*.

Although in many courses educators have faced issues with student disengagement and screens turned off, generally for this course screens were on, unless the students needed to leave the learning space for refreshments – perhaps an indication of the quality of investment and relationship.

Given the risk of disconnection in the virtual space, many new measures were put in place for this course. These included an additional question in the pre-course questionnaire regarding any student needs; highlighting psychological safety and thinking of ourselves as a team. We invited student agency by allowing them to decide who to speak next when introducing themselves and to consider their own boundaries through co-creating group rules. One of the easy wins in the online space is the presence of each person’s name on screen, allowing a mindful use of student names during group dialogue. Student tasks were carefully planned to cultivate connection and sense of safety – e.g. talking about something meaningful on their desk.

Modelling care was further enabled through flattening hierarchy to the point of vulnerable leadership [12], where educators create an open learning space by judiciously sharing of some of their own challenges – e.g. in clinical practice or online facilitation. This offers students permission to share their own vulnerable moments of learning.

### Dialogue

In online conversation, we were careful to cut back presentations to ten minutes or less, being aware of how disengaging online presentation of ideas can be. Instead, more active interaction of posing questions, facilitating dialogue, and engaging students in meaningful tasks was the focus across the course.

Specific attention was given to the loss of embodied and informal spaces supported by adding in extra breaks for movement away from the screen and the concept of kettle boiling. ‘Kettle boiling’ was created as a teaching strategy and a metaphor, emulating the time people spend around the kettle prior to a meeting getting a cup of tea or coffee. It involves putting participants in small groups with a very simple task, for example, to introduce themselves and talk about something interesting they learnt this week. This educational metaphor implies a ‘preparatory and warming up’ step to arrive fully into the learning process and connect with others, exercising their voice online. Further, student-led informal connection was recommended through WhatsApp groups.

Formal spaces for connection were supported by online collaboration tools such as Mentimeter to allow thinking alone – e.g. ‘how might the arts be of value to patients or to you as future clinicians?’, followed by conversations in groups of three and then large group discussion (i.e. a kind of ‘think-pair-share’). Use of Mentimeter or other online collaborative technologies such as Padlet, allowed rapid survey of the individual thinking across the whole class, promoting silent, anonymous time and space for more introverted participants to think and contribute to the subsequent group dialogue.

### Practice

Being creative and authentically sharing creative expression was central to growing trust and a sense of caring in the group. Some students valued being able to be creative in the privacy of their own room whilst online. Creating alone or simultaneously with others in the group enabled students to experience ‘flow’ [13] – total absorption in the present, highlighting one approach for self-care.

Sharing happened by holding up creations to the screen or by adding images to a Padlet site for all to view. Although not all students shared, some students during the pandemic added extra creations that they were doing outside of the course.

We encourage students to explore creative enquiry processes without worrying about outcomes, but more as a portrait of practice [14]. We explain that students may journey further in understanding when they are engaging with an arts-based medium which they are not familiar with.

Small and courageous risks are often taken by one student, enabling voice and agency for others as they use the creative process and products to metaphorically and sometimes explicitly express moments of personal reflection. This could also involve the holding of emotions. Although emotions and body language may be harder to read in the online space, creative exercises and expression enabled this dimension, alongside educational leadership that is unafraid of emotional moments. Negative emotions, for example, fear, guilt and shame expressed within a supportive environment can be powerfully transformative and allow honest conversation [15].

### Confirmation

**Figure 1:**
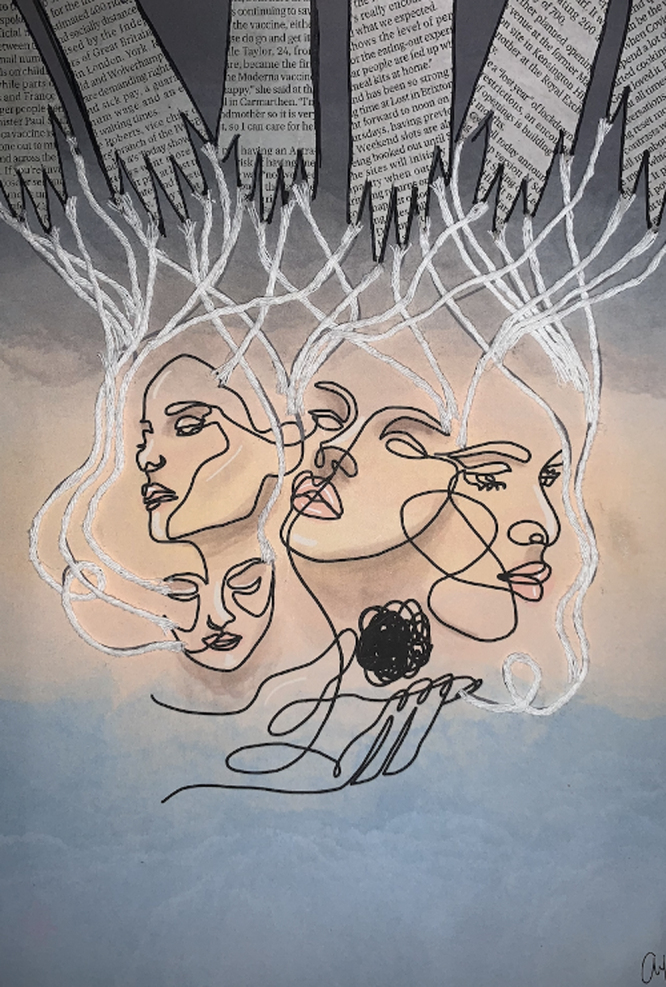
“Breaking Free from the Chains of Expectations” by Natasha Alia Razman, 2021.

During their final session, students confirm each other as each one presents their creative-reflective text relating to any dimension of medicine and healthcare. Presence, listening, curiosity and a beginners mind [16] enables sharing and learning to the point of transformation. Creating a confirmatory and compassionate space for exploration of lived experience in medical education is relevant to future understanding of practice as holistic and complex, built on intersubjective encounters between the selves of the patient or doctor. Figure 1 is an example of the final art piece, detailing the jagged hands as the voices that control us, the strings to the puppet faces that we put on to face the world, but underneath is a kind hand with the ball of wool and with it a choice to be kind to ourselves.

## Evaluation of Innovation

Our reflective analysis through self-study illuminates that it has been more than achievable to enliven a digital learning space, drawing on Noddings pedagogy of care to develop human dimensions and capabilities in medical students. In terms of impact on student engagement, feedback across all cohorts suggests the potential to facilitate a relational, transformative course in the online environment, for example:

‘I believe this [SSC] had life changing impact on how I see both my own personal life and medical career in the future…. Fostering a safe space for us to openly talk and communicate was essential in encouraging us to all feel comfortable in sharing opinions and deep thoughts with one and other. Speaking openly with my SSC group about many of the struggles of university life gave me a newfound confidence and hearing about shared worries helped to quell my own. I now felt much more in contact with these feelings and had the ability to share and convey these feelings with friends and family’. (First year male student, 2021)

What is striking is the lack of acknowledgement of student experiences being online. Students have developed the sense of ‘holding vulnerable spaces’ for each other, the transformative power of sharing in learning to build their confidence and sense of belonging. Further, when students did comment on the online learning space, it was to highlight the warmth and connection with colleagues.

‘I vividly recall being astounded at the support and warmth with which my peers approached my contribution, and this gave me a feeling of solidarity and acceptance that I had not yet felt during my experience of online medical school, and which boosted my confidence to contribute further in later discussion’. (First year female medical student, 2021)

## Critical Reflection

Our key findings point to the importance of values and approaches of the relational pedagogies; of setting the tone and crafting the digital learning space through care, compassion and vulnerability, allowing expression of emotion and experiences of human failure. Modelling care for our medical students, enabling dialogue and the practice of caring for each other, and also the confirmation of what each other bring, are multiple layers, all of which resonate with relational pedagogies [9] and accord with a social constructionist understanding of learning through encounter [17]. Perhaps not all of these layers are achievable in every session for students to develop and achieve. However, by reflecting on and designing for the learning across time and space, we can weave in these layers to support a direction of travel towards developing greater humanity in our students [18].

Limitations regarding this innovation include creative enquiry in medical education as potentially optional and niche, along with the cohort size being relatively small where bespoke care and attention might be given more readily, compared to large class settings. Additionally, it is important to note that the student interest for connection would have been heightened during the pandemic as many of them (at least first years) were learning from home and had not yet really had much experience of learning on campus and in-person. Further, the extra time and energy educators may need to invest in planning and facilitating human-centred digital learning sessions ought to be noted, to allow for the human dimension to flourish in a safe and productive manner. This might pose challenges for our increasingly time-poor educators teaching in a medical curriculum within our university systems. Finally, more research needs to be done to explore transferability of some of the approaches presented, to other medical and higher education contexts, including larger group setting and in compulsory rather than optional medical education spaces.

In conclusion, through the careful unpacking of Noddings’ conceptual framework illustrated by the case of creative enquiry course online, we have argued that planning and designing for the human dimension, consideration of the relational and how to support presence, connection and engagement are key aspects to productive teaching and learning online. Our case study of innovative approaches, underpinned by a theoretical framework of pedagogy of care demonstrates that such humanistic and relational pedagogies are possible to be designed for and essential to an increasingly digital, medical curriculum for the future.
